# Supplementary material for: Post-traumatic olfactory loss and brain response beyond olfactory cortex
Source: Sci Rep. 2021 Feb 17;11:4043. doi: 10.1038/s41598-021-83621-2 (PMC7889874; doi:10.1038/s41598-021-83621-2)

Supplementary Information for

Post-traumatic olfactory loss and brain response beyond olfactory cortex

Robert Pellegrino, Michael C. Farruggia, Dana M. Small, Maria G. Veldhuizen

Correspondence: Robert Pellegrino

Email: pellegrino.robert@gmail.com

Supplementary Table 1

Statistics of demographic and behavioral variables

	**normosmia**			**hyposmia**			**anosmia**			**test**	**statistic**	**overall p**	**normosmia vs hyposmia**	**normosmia vs anosmia**	**hyposmia vs anosmia**
**Variable**	mean	sd	normality	mean	sd	normality	mean	sd	normality						
**Duration impairment**				39.5	52.42	**<0.001**	32.34	37.88	**<0.001**	MW#	176.5	0.9262			
**Severity score**	0.563	1.153	**<0.001**	9.400	15.53	**0.0168**	18.79	18.77	0.1598	KW§	21.23	**<0.0001**	**0.0211**	**<0.0001**	0.2648
**Extent score**	0.375	0.719	**0.0065**	1.933	1.624	0.4498	3.263	1.968	**0.0069**	KW§	22.67	**<0.0001**	**0.0170**	**<0.0001**	0.2244
**age**	45.68	13.12	0.886	51.50	11.38	0.680	53.96	13.49	0.374	F*	2.496	0.091	0.1737	**0.0316**	0.5525
**threshold**	7.81	2.28	0.660	3.55	2.05	0.111	1.13	0.28	**<0.001**	KW§	50.38	**<0.001**	**0.011**	**<0.001**	**0.002**
**discrimination**	12.41	1.65	0.903	9.94	2.14	0.643	6.40	2.22	0.472	F	52.67	**<0.001**	**<0.001**	**<0.001**	**<0.001**
**identification**	13.55	1.22	0.408	8.56	2.45	0.513	3.96	1.79	0.119	F	162.6	**<0.001**	**<0.001**	**<0.001**	**<0.001**
**tdi**	33.77	3.08	0.799	22.06	3.73	0.593	11.49	2.83	0.790	F	290.7	**<0.001**	**<0.001**	**<0.001**	**<0.001**
**free identification**	0.90	0.18	**0.010**	0.39	0.35	0.386	0.07	0.15	**<0.001**	KW	43.64	**<0.001**	**0.0033**	**<0.0001**	**0.0221**
**intensity**	5.80	1.87	0.587	2.97	2.04	0.324	0.93	0.99	0.192	F	52.39	**<0.001**	**<0.001**	**<0.001**	**<0.001**
**pleasantness**	1.53	1.87	0.881	1.74	1.54	0.546	0.48	1.31	**0.029**	KW	7.352	**0.0253**	0.698	**0.0292**	**0.0169**
	**p(w)**	**p(m)**		**p(w)**	**p(m)**		**p(w)**	**p(m)**							
**gender**	0.68	0.32		0.38	0.63		0.68	0.32		Chi¶	4.665	0.097			

Mann-Whitney U test

*ordinary one-way ANOVA

§ Kruskal-Wallis ANOVA

¶ Chi-square test

Supplementary Table 2

Significant clusters of BOLD response to odor – odorless (regardless of group)

Label (Brodmann Area)	P_{FWE}	cluster size	T-value	x y z (MNI)
**Intra parietal sulcus**	**.003***	**243**	**5.620**	**-36 -60 54**
			3.565	-50 -60 48
			3.554	-42 -48 50
			3.338	-36 -64 40
**Cerebellum**	**.001***	**269**	**4.755**	**22 -76 -22**
			4.124	36 -74 -28
			3.697	46 -68 -28
			3.565	20 -86 -20
			3.321	24 -68 -28
			3.002	24 -88 -12
**Inferior frontal gyrus**	**<.001***	**328**	**4.695**	**-54 16 26**
			4.069	-52 12 42
			3.428	-44 20 30
			3.186	-54 20 10
			2.788	-50 8 24
**Posterior piriform**	**¶**	**44**	**4.053**	**-18 -8 -8**
			3.226	-20 -16 -12
**Anterior piriform**	**.007§**	**43**	**4.015**	**26 2 -20**
**Anterior piriform/ventral insula**	**¶**	**26**	**3.564**	**-32 8 -16**
**Posterior piriform**	**¶**	**7**	**3.000**	**22 -8 -14**

§ p-value for alpha = .05 FWE-corrected for multiple comparisons at the voxel level across a small volume search (SVC)

* p-value for alpha = .05 FWE-corrected for multiple comparisons at the cluster-level across the whole brain

¶ not significant corrected for multiple comparisons

Supplementary Table 3

Statistical values of equivalence tests of parameter estimates from peak voxels extracted from piriform clusters listed in Table 2 between the normosmia and anosmia groups

BRAIN REGION	TOST T-VALUE*	TOST P-VALUE
laPir	-4.784	0.000
raPir	-5.220	0.000
lpPir	4.241	0.000
rpPir	5.207	0.000

* TOST procedure based on Student's t-test based on equivalent bounds calculated from Han et al. 2018

Supplementary Table 4

Significant clusters of BOLD response to odor – odorless showing linear effect with olfactory function (by group)

Label (Brodmann Area)	P_{FWE}	CLUSTER SIZE	F-VALUE	X Y Z (MNI)
**Anterior insula**	**.001§**	**77**	**4.497**	**44 14 6**
**Posterior cingulate gyrus**	**.042***	**108**	**4.170**	**0 -22 48**
			3.855	-12 -28 44
			3.365	6 -28 48
**Ventromedial prefrontal gyrus**	**<.001***	**324**	**3.995**	**2 46 -6**
			3.525	-2 56 -4
			3.257	6 54 10
			3.061	-2 58 4
			2.934	10 44 -2
**Anterior insula**	**.031§**	**50**	**3.619**	**-34 16 10**
**Medio dorsal thalamus**	**.017§**	**22**	**3.530**	**-6 -22 8**
**Medio dorsal thalamus**	**.038§**	**22**	**3.252**	**10 -18 10**

§ p-value for alpha = .05 FWE-corrected for multiple comparisons at the voxel level across a small volume search (SVC)
* p-value for alpha = .05 FWE-corrected for multiple comparisons at the cluster-level across the whole brain

Supplementary Table 5. Top 10% high-degree nodes obtained from CPM analysis

NODE	DEGREE	ANATOMICAL LABEL	COORDINATES (MNI)	LOBE	NETWORK
**220**	7	Ventral Anterior Cingulate	-3.83,-5.05,32.6	Limbic	Limbic
**218**	6	Premotor + Supplementary Motor	-7.75,-22.37,46.05	Limbic	Motor
**39**	6	Primary Sensory	20.01,-33.24,69.77	Parietal	Motor
**260**	5	Caudate	-14.6,-3.51,21.09	Subcortical	Basal Ganglia
**216**	5	Primary Visual	-22.11,-66.7,7.45	Occipital	Visual I
**197**	5	Superior Temporal Gyrus	-57.05,-14.52,-6.87	Temporal	Motor
**188**	5	Temporal Pole	-49.73,6.39,-15.15	Temporal	Motor
**132**	5	Brainstem	6.31,-24.92,-17.47	Brainstem	Cerebellum
**15**	5	Frontal Eye Fields	6.69,21.42,31.46	Prefrontal	Limbic
**234**	4	Parahippocampus	-30.54,-23.92,-26.61	Limbic	Basal Ganglia
**227**	4	AgrRetrolimb?	-7.47,-42.12,13.32	Limbic	Default Mode
**221**	4	Ventral Anterior Cingulate	-5.1,13.15,28.74	Limbic	Limbic
**187**	4	Temporal Pole	-49.49,11.11,-30.56	Temporal	Medial Frontal
**163**	4	Premotor + Supplementary Motor	-56.98,-3.43,6.82	Motor Strip	Motor
**162**	4	Premotor + Supplementary Motor	-9.06,0.38,66.53	MotorStrip	Medial Frontal
**160**	4	Premotor + Supplementary Motor	-16.2,-19.23,69.53	MotorStrip	Motor
**150**	4	Frontal Eye Fields	-5.03,17.67,46.05	Prefrontal	Medial Frontal
**145**	4	dlPFC (dorsal)	-10.15,55.69,30.24	Prefrontal	Medial Frontal
**125**	4	Putamen	14.03,8.29,-9.5	Subcortical	Basal Ganglia

**84**	4	Ventral Anterior Cingulate	5.25,-1,35.56	Limbic	Motor
**69**	4	Fusiform	55.22,-56.3,-4.78	Temporal	Visual Association
**44**	4	Visual Motor	7.48,-57.28,61.79	Parietal	Limbic
**42**	4	Visual Motor	14.82,-68.41,34.88	Parietal	Visual I
**34**	4	Insula	41.79,4.97,-7.62	Insula	Motor
**5**	4	Ant PFC	8.18,45.93,-1.72	Prefrontal	Default Mode
**261**	3	Putamen	-24.78,5.62,-0.08	Subcortical	Basal Ganglia
**248**	3	Cerebellum	-8,-68.4,-19.85	Cerebellum	Cerebellum

Supplementary Figure 1. Participant brain trauma severity score and extent score. Left panel shows mean (center line) severity score with standard error of the mean bars (shorter upper and lower bars), with individual data points overlaid for normosmia (solid black squares), hyposmia (open gray circles), and functional anosmia (open black diamonds) participant groups. Severity score reflects the severity of the damage. Right panel shows the mean extent score, with higher numbers indicating more brain regions affected, regardless of how severe the damage was within each brain regions. Bracket indicates significant post-hoc t-test between groups (corrected for multiple comparisons) with p-value.

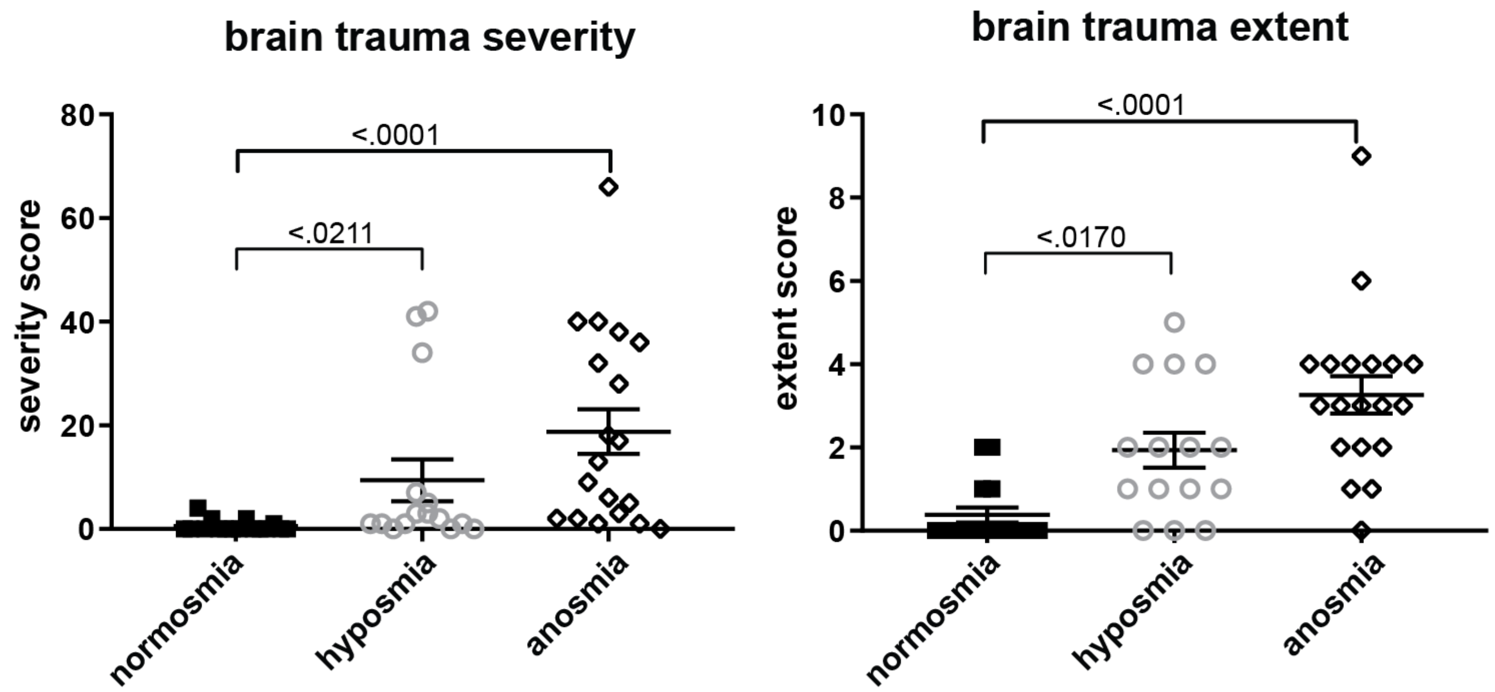

Supplement: Supplementary file 1 — Supplementary Information [file 41598_2021_83621_MOESM1_ESM.pdf]
